# Comparing the Predictive Power of Preoperative Risk Assessment Tools to Best Predict Major Adverse Cardiac Events in Kidney Transplant Patients

**DOI:** 10.1155/2019/9080856

**Published:** 2019-03-20

**Authors:** Colin P. Dunn, Emmanuel U. Emeasoba, Ari J. Holtzman, Michael Hung, Joshua Kaminetsky, Omar Alani, Stuart M. Greenstein

**Affiliations:** ^1^Department of Surgery, Albert Einstein College of Medicine, 10461 Bronx, NY, USA; ^2^The Montefiore Einstein Center for Transplantation, Montefiore Medical Center, Bronx, 111 East 210^th^ Street, 10467 NY, USA

## Abstract

**Background:**

Patients undergoing kidney transplantation have increased risk of adverse cardiovascular events due to histories of hypertension, end-stage renal disease, and dialysis. As such, they are especially in need of accurate preoperative risk assessment.

**Methods:**

We compared three different risk assessment models for their ability to predict major adverse cardiac events at 30 days and 1 year after transplant. These were the PORT model, the RCRI model, and the Gupta model. We used a method based on generalized U-statistics to determine statistically significant improvements in the area under the receiver operator curve (AUC), based on a common major adverse cardiac event (MACE) definition. For the top-performing model, we added new covariates into multivariable logistic regression in an attempt to create further improvement in the AUC.

**Results:**

The AUCs for MACE at 30 days and 1 year were 0.645 and 0.650 (PORT), 0.633 and 0.661 (RCRI), and finally 0.489 and 0.557 (Gupta), respectively. The PORT model performed significantly better than the Gupta model at 1 year (*p*=0.039). When the sensitivity was set to 95%, PORT had a significantly higher specificity of 0.227 compared to RCRI's 0.071 (*p*=0.009) and Gupta's 0.08 (*p*=0.017). Our additional covariates increased the receiver operator curve from 0.664 to 0.703, but this did not reach statistical significance (*p*=0.278).

**Conclusions:**

Of the three calculators, PORT performed best when the sensitivity was set at a clinically relevant level. This is likely due to the unique variables the PORT model uses, which are specific to transplant patients.

## 1. Introduction

Cardiovascular disease (CVD) is the most common cause of death after successful renal allograft transplant. Many studies have shown that renal transplant recipients have an increased risk of CVD over the general population. Because transplant recipients are uniquely at risk, they require accurate prediction of their cardiovascular fitness before undergoing transplant surgery. There are many risk assessment calculators created for general surgery patients, but they may not function in transplant patients. For example, the Framingham risk calculator consistently underpredicts adverse cardiovascular events after transplant [1, 2]. The three calculators in this study were chosen because they are commonly used and/or were designed with kidney transplant recipients in mind. The Patient Outcomes in Renal Transplant (PORT) risk assessment calculator was created specifically for kidney transplant with data from 14 transplant centers worldwide. In the PORT study, the overall C-statistic for this calculator was 0.80–0.85 [3, 4]. The Revised Cardiac Risk Index (RCRI) was also shown to predict cardiovascular complications in kidney transplant recipients with a C-statistic of 0.77 [5]. There is also another cardiac risk assessment tool developed by Gupta et al. which had a C-statistic of 0.874, but was not created with data from transplant patients, although it did outperform the RCRI when used for nontransplant operations [6]. None of these three calculators have been compared against each other within the same cohort. For this reason, we decided to compare the utility of the three different CV risk calculators using data from our own transplant center, to assess which of the models most accurately predicts long-term and short-term Major Adverse Cardiac Events (MACE).

## 2. Materials and Methods

### 2.1. Data Acquisition

After IRB approval was obtained (#2014-3329), we utilized the transplant database at Montefiore Medical Center to identify all adult patients who were transplanted from 2005 to 2010 at our center from a living or cadaveric donor. Patient data were obtained through Clinical Looking Glass (a software/database combination for Montefiore Health System), the Montefiore Transplant Database, and direct review of the electronic medical record [7]. Death was determined via in-house medical records or Social Security Death Index.

### 2.2. Variables within Each Calculator

The PORT model is composed of the following variables: age, sex, history of diabetes, history of cancer, donor type (living or deceased), years from end-stage renal disease to transplant, and the number of cardiovascular comorbidities. These comorbidities were defined as previous myocardial infarction (MI), congestive heart failure (CHF), coronary revascularization, cerebrovascular accident (CVA), or peripheral arterial disease (PAD) surgery. The RCRI is composed of coronary artery disease, CHF, cerebrovascular disease, insulin-dependent diabetes mellitus, serum creatinine more than 2 mg/dl, and high-risk (suprainguinal, vascular, and abdominal) surgery. The Gupta model is composed of age at time of procedure, whether the preoperative creatinine was >1.5 mg/dl, the ASA class, the general preoperative functional status, and the category of procedure (peripheral vascular for this cohort). General functional status was recorded by nurses as part of routine preoperative assessment at our institution.

### 2.3. MACE Definitions

The RCRI calculator defined MACE as MI, pulmonary edema, ventricular fibrillation, primary cardiac arrest, or complete heart block [8]. The Gupta calculator defined MACE as MI or cardiac arrest [6]. The PORT calculator defined MACE as fatal or nonfatal MI, angioplasty or stenting, or sudden death [4].

### 2.4. Additional Definitions

Clinical Looking Glass defines Socioeconomic Status as a numeric value based on the median household income, median value of housing, percent of households receiving interest, net rental income, education, percentage of adults who completed college, and percentage of adults employed in executive, managerial, or professional positions within the same neighborhood or zip code as the patient. Peripheral vascular disease was defined as any documentation of claudication in the past year before transplant, any inpatient admission due to peripheral vascular disease at any point before transplant, or any peripheral revascularization procedure at any point before transplant. Coronary revascularization history was defined as any stent placement, angioplasty, or other revascularization procedure at any time prior to transplant. Ischemic heart disease, stroke, and diabetes were each defined as documentation via ICD-9 code in inpatient, outpatient, or emergency department visit settings, or any addition of the disease to the problem list.

Patients were considered lost to follow-up by 30 days if there was no record of their death and no physician encounter between their discharge and 90 days after transplant. The physician encounter could be any type of surgery, a visit to an ambulatory clinic of any kind, emergency department visit (seen by ED physician), or any form of inpatient hospitalization. Patients were considered lost to follow-up by 1 year if there was no record of their death, and they did not see a physician in our network within 6 months prior to their one-year after transplant anniversary date. Patients deemed lost to follow-up were excluded from data analysis.

### 2.5. Statistical Testing

A *p* value of 0.05 or less was considered statistically significant. All confidence intervals were 95% and all tests were two-tailed unless otherwise noted. Categorical variables were described using absolute numbers and percentages. Continuous variables were described using the mean and standard deviation or the median if the data were skewed. All analyses were performed using R, the open-source statistical computing software [9]. Tables were created using the “tableone” package [10]. The “pROC” package was used to graph receiver operator curves, compute the AUC (area under the receiver operator curve), and create confidence intervals around the graph [11]. Missing data were managed via listwise deletion.

**Figure 1:**
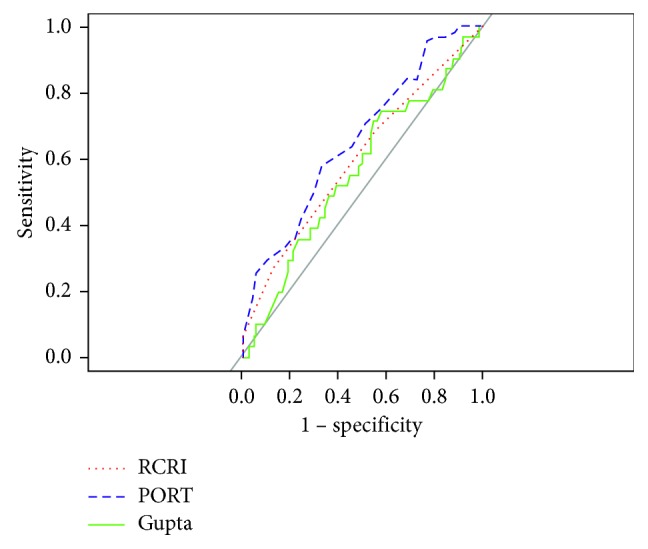
Comparing ROC curves on each model 1 year after transplant, using Gupta definition of Major Adverse Cardiac Event.

In order to determine statistically significant differences in AUC, the methods of Delong et al. were used to create a covariance matrix for each receiver operator curve (ROC) [12]. When comparing calculators based on a fixed sensitivity, a bootstrapping method with 2000 replications was used, as described by Pepe et al. [13]. These bootstrapping tests were one-tailed. The direction of the one-tailed test was determined after visual inspection of the graph of all three ROC's together (Figure 1).

In order to improve upon the existing calculators, a new calculator was created to predict MACE at 1 year after transplant. The following covariates were entered into a multivariable logistic regression model a priori: socioeconomic status (SES), body mass index (BMI), race, ethnicity, the Charlson Comorbidity Index, preoperative functional status, ASA class, history of CHF, and whether there had been a previous transplant. All covariates with a resulting *p* value of 0.05 or less were included into the new calculator.

## 3. Results

### 3.1. Study Population

No patients were lost to follow-up at the 30-day mark and thirty-six patients (6.68%) were considered lost to follow-up at the 1 year mark based on our definitions above. After excluding patients lost to follow-up and 45 pediatric recipients, there were 503 patients in our cohort. There was no missing data in either the predictors of MACE in each calculator, or the MACE outcomes themselves. Our cohort was 35.8% Black, 26.4% Multiracial, 17% White, and 22.6% Declined to state/Other. There were 294 (58.4%) male patients and 209 (41.6%) females. The median age was 52. Forty-one percent of our cohort had diabetes mellitus. Forty-five (8.9%) patients were repeat transplants; 68.2% of our cohort received cadaveric transplants, and 31.8% received living grafts. Additional demographic data are available in Table 1.

### 3.2. Adverse Cardiac Events

Within one year of transplant, four patients required a coronary revascularization procedure, of which one required two separate coronary revascularization procedures. Ten patients had an MI, 31 patients had an adverse event according to Gupta, 42 patients had a MACE by PORT criteria, and 93 patients had a MACE by RCRI. There were 23 patients (4.57%) who died within 1 year of transplant. We were able to determine cause of death for 13 of the 23 since they occurred in house. Of these, 6 were secondary to cardiovascular disease, five were secondary to infection, one was secondary to an adverse reaction from a medication, and one was secondary to bleeding.

### 3.3. Comparing Model Performance at 30 Days and 1 Year

**Figure 2:**
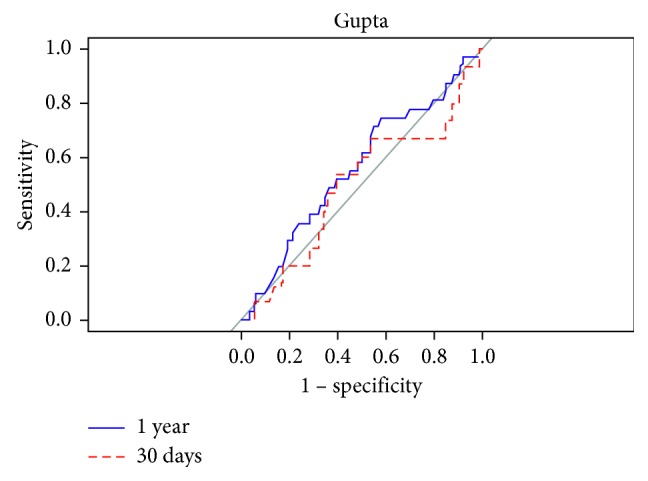
Comparing predictability of major adverse cardiac events at 30 days and 1 year using the Gupta calculator.

**Figure 3:**
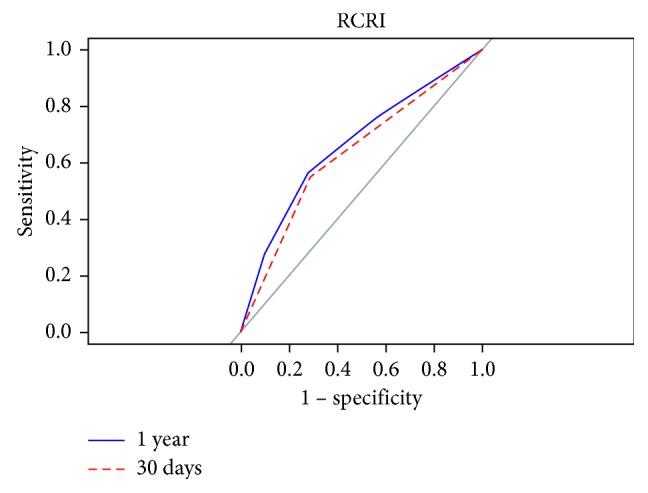
Comparing predictability of major adverse cardiac events at 30 days and 1 year using the RCRI calculator.

**Figure 4:**
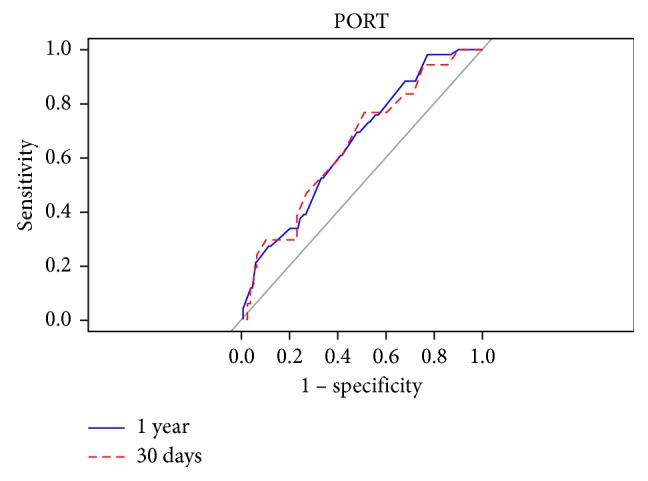
Comparing predictability of major adverse cardiac events at 30 days and 1 year using the PORT calculator.

We created ROC curves for each calculator predicting their respective definitions of MACE at 30 days and 1 year after transplant (Figures 2–4). The AUC for Gupta was 0.489 and 0.557 at 30 days and one year, respectively. The AUCs for PORT at 30 days and 1 year after transplant were 0.645 and 0.650, respectively. The AUCs for the RCRI at 30 days and 1 year were 0.633 and 0.661, respectively. A comparison between the 30-day and 1 year marks within each calculator did not reach statistical significance (Gupta *p*=0.499, RCRI *p*=0.611, and PORT *p*=0.954).

### 3.4. Comparing between Models at 1 Year

Because a statistically significant difference between AUC at 30 days and AUC at 1 year was not detected, all further analyses were conducted at 1 year after transplant. Direct statistical comparison of the predictive capacity of each calculator required a standardized outcome. We used the Gupta definition for MACE because it was the most specific to cardiovascular pathology (see Materials and Methods). We used one-tailed tests after graphing the three receiver operator curves together (Figure 1). At one year after transplant, there was no statistically significant difference between PORT and RCRI (*p* value = 0.089) or Gupta and RCRI (*p* value = 0.281). However, there was a statistically significant difference between PORT and Gupta (*p*=0.039), meaning that the AUC for PORT was significantly greater than the AUC for Gupta.

To evaluate the calculators in a clinically relevant way, the specificity of each calculator was compared when the sensitivity was set at 95%. PORT outperformed Gupta, with a specificity of 0.227 versus 0.080, respectively (*p*=0.017). Additionally, PORT outperformed RCRI, with specificities of 0.227 and 0.071, respectively (*p*=0.009). The comparison between RCRI and Gupta did not reach statistical significance (specificity 0.071 and 0.080, *p*=0.557).

### 3.5. Improving Upon the Existing PORT Model

After establishing that the PORT model performed better than the Gupta and RCRI at a high sensitivity, we attempted to improve upon it with additional covariates (see Materials and Methods). After creating the model, there were 21 patients (4.17%) from this group who had missing data and hence were subject to listwise deletion. None of the covariates met our *p* value threshold of less than 0.05 for significance and as such we were unable to improve the PORT model.

## 4. Discussion

In this retrospective observational study, we identified the best predictor of cardiovascular complications following renal transplantation. Other studies have made extensive comparisons of surgical risk assessment for general surgery patients. One of the most extensive reviews compared 27 predictors of postoperative surgical risk [14]. Other studies have conducted similar comparisons on a smaller scale using fewer risk assessment tools [15–18]. Still other studies have compared cardiovascular-specific risk across several assessment tools [19], but no such study has been conducted specifically for kidney transplant surgery. For this reason, we compared three of the most commonly used cardiac risk assessment models to anticipate adverse cardiac events following kidney transplant [4, 6, 8].

Of the three risk-assessment tools, only PORT was developed specifically for postoperative cardiovascular risk in renal transplant patients [4]. RCRI and Gupta were developed to assess risk for a broader range of surgical interventions [6, 8]. Some of the predictors were homogenous throughout our cohort, such as the most recent serum creatinine being >1.5, or the type of surgery being performed. Because these predictors did not vary, they were not helpful in distinguishing which patient would have an adverse cardiac event. It is therefore logical that PORT would emerge as the superior model. Furthermore, the added covariates from the Gupta and RCRI models failed to reach our threshold *p* value of 0.05 for inclusion into an enhanced model. This suggests that the covariates, which were predictive of MACE within RCRI and Gupta, were likely the covariates, which were already within PORT.

The Gupta definition of MACE was used when comparing the discriminative power of the three calculators in order to provide a standardized outcome [6]. It is likely that our study design was therefore biased in favor of Gupta since the other two models were not designed specifically to detect our working definition of MACE. Despite this advantage, PORT still outperformed Gupta, as well as RCRI, thereby supporting our conclusion that PORT is indeed a superior model for MACE.

It is interesting to note the difference in findings between analyzing the receiver operator curves in their entirety versus the right-most portions. While statistically significant, PORT's superiority is less overwhelming when the entire ROCs are compared to one another. PORT's discriminative ability is greatest toward the right-most portion of the ROC where sensitivity of cardiac risk prediction is high. We believe these prediction tools should be used at a high sensitivity, given the morbidity and mortality associated with major adverse cardiac events. The cutoff points of these assessment tools should be set such that the vast majority of high-risk patients are identified, even if a high sensitivity results in more false positives.

Despite our determination that history of stroke and living versus cadaveric source of kidney were significant when analyzed alone, their incorporation into a modified PORT model did not result in a statistically significant improvement over the original PORT. An explanation for this is that these factors are too similar to covariates already present in the original PORT. For example, if the cadaveric versus living source of the kidney already strongly correlates with estimated GFR or rate of acute rejection, its addition into the model would not result in significance.

There are some limitations to our study. First, our sample size was smaller than the studies, which produced the original three assessment tools. However, while larger sample sizes can better estimate effect size, our study nonetheless produced statistically significant results. A second limitation is the necessity to standardize our study on a single definition of MACE when comparing the different risk calculators. While PORT's superiority may in fact be understated by our use of MACE according to Gupta, perhaps defining MACE according to RCRI could yield different results. Finally, we did have a small amount of missing data and some patients who were lost to follow-up. However, the data appeared to be missing at random and therefore would not unfairly alter the strength of association between certain covariates and MACE.

## 5. Conclusion

We have demonstrated the different models to predict MACE at one year following transplant. However, there is need for a prospective study to further evaluate the effectiveness of the three assessment tools. To truly know your risk, bear in mind the risks of your patient population.

## Figures and Tables

**Table 1:** Demographic data.

*n*	503
Age at transplant (median [IQR])	52.00 [42.00, 61.00]
Gender = male (%)	294 (58.4)
Race (%)	
Black or African American	180 (35.8)
Declined/Other	111 (22.1)
Multiracial	133 (26.4)
White	79 (15.7)
Ethnicity (%)	
Declined	23 (4.57)
Hispanic or Latino	205 (40.8)
Not Hispanic or Latino	275 (54.7)
SES (median [IQR])	−2.48 [−5.56, −0.90]
Donor living or cadaveric = living (%)	160 (31.8)
Previous transplant = yes (%)	45 (8.9)
BMI (mean (sd))	27.14 (5.38)
Diabetes = yes (%)	207 (41.2)
Insulin = yes (%)	143 (28.4)
Years on dialysis (median [IQR])	3.81 [1.03, 7.01]
MI before transplant = yes (%)	50 (9.9)
Peripheral arterial disease surg. = yes (%)	37 (7.4)
Cancer = yes (%)	22 (4.4)
Charlson Score (median [IQR])	0.00 [0.00, 2.00]

BMI = body mass index; MI = myocardial infarction; SES = socioeconomic status; SD = standard deviation; IQR = interquartile range.

## Data Availability

The data used to support the findings of this study have not been made available because of patient confidentiality.

## Abbreviations

AUC: Area under the receiver operator curve
ROC: Receiver operator curve
PORT: Patient outcomes in renal transplant
RCRI: Revised Cardiac Risk Index
ASA: American society of anesthesiologists
MACE: Major adverse cardiac event
MI: Myocardial infarction.
